# The Dietary Phytochemical Index Is Inversely Associated With the Odds of Premature Coronary Artery Disease (PCAD) in Iranian Adults: Iran Premature Coronary Artery Disease (IPAD) Study

**DOI:** 10.1002/fsn3.70943

**Published:** 2025-09-26

**Authors:** Motahare Bateni, Ehsan Zarepur, Fahimeh Haghighatdoost, Noushin Mohammadifard, Minoo Dianatkhah, Alireza Khosravi Farsani, Nahid Azdaki, Nahid Salehi, Masoud Lotfizadeh, Samad Ghaffari, Arsalan Salari, Mostafa Cheraghi, Ahmadreza Assareh, Mehrnoosh Arefianjazi, Nizal Sarrafzadegan

**Affiliations:** ^1^ Isfahan Cardiovascular Research Center, Cardiovascular Research Institute Isfahan University of Medical Sciences Isfahan Iran; ^2^ Interventional Cardiology Research Center, Cardiovascular Research Institute Isfahan University of Medical Sciences Isfahan Iran; ^3^ Hypertension Research Center, Cardiovascular Research Institute Isfahan University of Medical Sciences Isfahan Iran; ^4^ Iranian Network of Cardiovascular Research (INCVR) Tehran Iran; ^5^ Cardiovascular Diseases Research Center Birjand University of Medical Sciences Birjand Iran; ^6^ Clinical Research Development Unit, Razi Hospital Birjand University of Medical Sciences Birjand Iran; ^7^ Cardiovascular Research Center, Health Institute Kermanshah University of Medical Sciences Kermanshah Iran; ^8^ Department of Community Health Shahrekord University of Medical Sciences Shahrekord Iran; ^9^ Cardiovascular Research Center Tabriz University of Medical Sciences Tabriz Iran; ^10^ Cardiovascular Diseases Research Center, Department of Cardiology, Heshmat Hospital, School of Medicine Guilan University of Medical Sciences Rasht Iran; ^11^ Department of Cardiovascular Research Center, Shahid Rahimi Hospital Lorestan University of Medical Sciences Khorramabad Iran; ^12^ Atherosclerosis Research Center Ahvaz Jundishapur University of Medical Sciences Ahvaz Iran; ^13^ Department of Natural Sciences, School of Science and Technology University of Georgia Tbilisi Georgia

**Keywords:** cardiovascular disease, dietary phytochemical index, phytochemical, plant foods, premature coronary artery disease (PCAD)

## Abstract

There is no evidence to prove the relationship between Dietary Phytochemical Index (DPI) and the incidence of premature coronary artery disease (PCAD) among the Iranian population. Therefore, this research investigates the association between the DPI score and the odds of PCAD within a nationally representative Iranian population. This case‐control study was performed on 3161 (*n* = 2006 case and *n* = 1155 control) Iranian population < 70 y in women and < 60 y in men. Subjects showing normal results on coronary angiography comprised the control group, and those with stenotic lesions exceeding 75% in at least one coronary artery or over 50% in the left main coronary artery were identified as the case group. Information on dietary intake was collected using a validated 110‐item food frequency questionnaire. Using the McCarthy equation, the DPI was calculated. A binary logistic regression model was used to evaluate the association between DPI and PCAD. The mean age of the study population was 53.7 (SD = 7.6) years, and 45.1% of them were female. In the unadjusted model, individuals in the highest quartile of DPI exhibited a significantly reduced likelihood of developing PCAD compared to those in the lowest quartile (OR = 0.25, 95% CI: 0.20–0.31, *P* trend < 0.001). Adjustment for potential confounders further reinforced the relationship, indicating that participants in the top DPI quartile had 89% reduced odds of PCAD relative to those in the first quartile (95% CI: 0.08–0.17, *P* trend < 0.001). Our findings revealed a significant negative association between the DPI and the odds of PCAD. However, more research is necessary to determine this association.

## Graphical abstract

The association between Dietary Phytochemical Index and the risk of premature coronary artery disease.

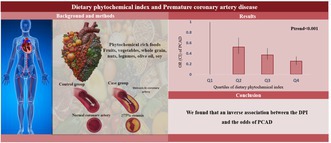

## 1 Introduction

Coronary artery disease (CAD) is an atherosclerotic disease characterized by inflammation (Ross 1999). Despite its impact across all age groups, there is a significant upward trend in CAD prevalence among younger individuals (Poorzand et al. 2019). Premature coronary artery disease (PCAD) is defined by its occurrence before the age of forty‐five in males and fifty‐five in females, though the age cut‐off may vary between 45 and 65 years (Mohammad et al. 2015; Khot et al. 2003; Achari and Thakur 2004; Panwar et al. 2011). Approximately 4 to 10% of CAD cases affect individuals younger than 45 years (Mohammad et al. 2015). PCAD is growing in developing countries like Iran (Sarrafzadegann et al. 2009). It is concerning compared to CAD because it belongs to the younger age group and imposes a heavier burden on the family and society (Wang et al. 2020). Consequently, the prevention of PCAD is of great importance and relies on lifestyle modification. One of the crucial factors to prevent PCAD is to modify the diet and follow a healthy eating pattern (Osadnik et al. 2018).

Evidence from prior research suggests that both plant‐based and Mediterranean diets may contribute to reducing the risk of cardiovascular disease (Salas‐Salvadó et al. 2018; Hemler and Hu 2019; Patel et al. 2017). Also, previous investigations have elucidated the relevance of various food groups, notably nuts, fruits, vegetables, whole grains, legumes, and olive oil, recognized for their abundant reservoirs of phytochemicals. These bioactive compounds, comprising isoprenoids, phenolic compounds such as flavonoids, lignans, and phenolic acid, organosulphur compounds like isothiocyanate, and allyl sulfurs, are derived from plants and endowed with non‐nutritive properties.

These phytochemical compounds have been linked to a reduced risk of developing PCAD and other cardiovascular events (Martínez‐González et al. 2015; Azadbakht et al. 2019). The potential mechanisms underlying the cardioprotective effects of a diet rich in phytochemicals might be reducing inflammation, oxidative stress, maintaining vascular function, and lowering the risk of obesity (Vincent et al. 2010; Esposito et al. 2004; Keaney Jr et al. 2003).

Based on the recognized health effects of phytochemicals, McCarty formulated the Dietary Phytochemical Index (DPI), defined as the ratio of daily energy intake contributed by foods containing phytochemicals (McCarty 2004).

Although the DPI has some limitations, such as variability in phytochemical content based on growing conditions, ripeness, and preparation methods, it can be used as a diet quality index (do Nascimento Nunes 2008). Various studies have explored the relationship between DPI and chronic diseases. For example, these studies have revealed an inverse association between DPI and stroke (Rigi et al. 2022), dyslipidemia (Golzarand et al. 2014), oxidative stress (Hamedi‐Shahraki et al. 2023), breast cancer (Acikgoz Pinar et al. 2023), metabolic syndrome (Dehghani Firouzabadi et al. 2021), hypertension (Golzarand et al. 2015), diabetic nephropathy (Bahrampour et al. 2023), and non‐alcoholic fatty liver disease (NAFLD) (Ahmadi et al. 2023) but some studies have reported no significant association between DPI and metabolic syndrome and various cardiometabolic risk factors (Hamedi‐Shahraki et al. 2023; Dehghani Firouzabadi et al. 2021; Golzarand et al. 2014). Although numerous studies have examined CVD risk factors, no research has evaluated the relationship between DPI and CVD events specifically in the Iranian adult population. Moreover, given that the results of the available studies on cardiometabolic risk factors are controversial and considering the relevance of the incidence of PCAD at a young age, the current case–control research aimed to evaluate the association of DPI with PCAD among Iranian adults.

## 2 Methods

### 2.1 Study Population

The study design is a non‐match case–control. This investigation was carried out as part of the Iran Premature Coronary Artery Disease study (IPAD). IPAD is a multi‐center research involving nine different Iranian ethnicities aimed at determining potential risk factors of PCAD. Sample size estimation was performed using a 5% alpha level, 80% power, and an expected odds ratio of 1.30, using the suggested formula for case–control studies. Given a 30% prevalence of PCAD, P2 is set at 0.3 and the odds ratio at 1.3 (as determined for the main sample size in the original study). P1 is calculated using P2, and then the sample size is estimated by the recommended formula for case–control studies. Based on this, the sample size obtained for each group is 524 participants. Considering potential data loss, an additional 20% is added, resulting in 628 participants per group to ensure adequate power for the study.

Our sample included 3293 patients. The convenience sampling method was employed to select study participants, and they were selected from among those who attended for coronary angiography in reference hospitals. For more clarity, we stated a representative sample of Iranians who attended for angiography. The overall sample was proportionally allocated based on the distribution of the different ethnic groups. This study was conducted on females under the age of 70 and males under the age of 60 years who underwent coronary angiography. In this study, a wider range was considered due to the limitation for the enrollment of participants. Our criterion for the case group was ≥ 75% stenosis in at least one single coronary artery or a stenosis of ≥ 50% in the left main coronary artery (González et al. 2005). Patients with normal findings on coronary angiography comprised the control group. Individuals with any prior history of coronary artery disease, including procedures like coronary bypass surgery, percutaneous coronary intervention, and balloon angioplasty, were excluded. Participants reporting extreme values for daily energy intakes above 4200 or below 800 kcal/day (Fung et al. 2002) were excluded. The final study population consisted of 3293 individuals. After exclusions, 3161 participants (*n* = 2006 case and *n* = 1155 control) remained. Ethical approval was granted by the Research Ethics Committee of Isfahan University of Medical Sciences (IR.MUI.REC.1396.2.055). All participants provided written informed consent, and the study adhered to the Declaration of Helsinki guidelines.

### 2.2 Data Collection

Trained interviewers collected demographic and lifestyle data including age, sex, marital status, education, physical activity, smoking, alcohol drinking, and dietary intake. The classification of education includes undergraduate (under bachelor's education), graduate (bachelor's education), and post‐graduate (master's education and above). Physical activity and dietary intake were investigated by a Persian, validated version of the International Physical Activity Questionnaire (IPAQ) (Moghaddam et al. 2012). Weight and height were measured following standard World Health Organization (WHO) protocols (World Health Organization 1989). The patient's medical history was also recorded. Diabetes mellitus (DM) was defined as either the use of antidiabetic medications or a fasting blood glucose level ≥ 126 mg/dL. Hypertension (HTN) was defined as the use of antihypertensive drugs, systolic blood pressure (SBP) ≥ 130 mmHg, or diastolic blood pressure (DBP) ≥ 80 mmHg. Dyslipidemia was defined as having low‐density lipoprotein (LDL) ≥ 130 mg/dL, total cholesterol > 200 mg/dL, triglycerides ≥ 150 mg/dL, or high‐density lipoprotein (HDL) < 40 mg/dL in men or < 50 mg/dL in women (Expert Panel on Detection E and Treatment of High Blood Cholesterol in A 2001).

### 2.3 Dietary Assessment

Participants' dietary habits over the preceding year were recorded through a 110‐item FFQ questionnaire, validated in Persian (Mohammadifard et al. 2021). The FFQ had been validated earlier within the Iranian population, demonstrating moderate agreement with 24‐h dietary recalls for macronutrients (e.g., *r* = 0.48 for protein), which supports its acceptable level of validity. The FFQ assessed food intake frequency applying common portion sizes for each food item, with a frequency of never/rarely to ≥ 6 times/day. Portion sizes reported by participants were translated into grams based on standard household measurements. Subsequently, mean values for energy and nutrient intake were estimated utilizing a version of Nutritionist IV software specifically adapted for Iranians.

### 2.4 DPI Calculation

DPI was determined by the McCarty method (McCarty 2004), employing the formula: (energy intake from phytochemical‐rich foods/total energy) × 100. Phytochemical‐rich foods include fruits, natural fruit and vegetable juices, vegetables, tomato sauce, legumes, nuts, whole grains, soy products, and olive oil. Potatoes were excluded from the DPI calculation because their phytochemical content is relatively low compared to other vegetables.

### 2.5 Statistical Analysis

All statistical analyses were done using SPSS statistics software (version 25; SPSS Inc). The quartiles of DPI were determined. Comparisons of participants' characteristics across the DPI quartiles were conducted by the chi‐square test for categorical variables and ANOVA for continuous variables. Categorical and continuous variables were reported as frequency (*n*, %) and mean ± standard deviation (SD), respectively. Dietary intake comparisons across DPI quartiles were analyzed by analysis of covariance (ANCOVA), adjusted for age, sex, and daily energy intake. We used binary logistic regression to evaluate the risk of PCAD across the quartiles of DPI in different statistical models. The crude model was unadjusted, while model 1 was adjusted for sex, age, and daily energy intake. Model 2 was further controlled for smoking, marital status, education, physical activity, and alcohol, and model 3 was further adjusted for dietary intake including high‐fat dairy, low‐fat dairy, red and processed meat, potato, fiber, refined grain, and sugar‐sweetened beverages. Finally, model 4 was additionally adjusted for BMI, diabetes mellitus, hypertension, dyslipidemia, and Aspirin. STATA was used to perform dose–response analysis. Restricted cubic spline was used to show the possible nonlinear dependency of the association between DPI and odds of PCAD, using three knots according to the percentiles of DPI, 30%, 60%, and 90%. A threshold of *p* value < 0.05 was used to determine significance.

## 3 Results

A total number of 3161 participants was included in this study. The mean age of participants was 53.7 (SD = 7.6) and 45.1% of participants were women. The mean ± SD age of case and control groups was 54.71 ± 7.14 and 51.96 ± 8.23, respectively (p value < 0.001). Also, 657 (27.7%) of participants in the case group and 727 (55%) of participants in the control group were women (p value < 0.001). The range of DPI in this study was from 2.71 to 67.82, with a mean of 22.06 (SD = 9.0). The range of DPI in the first, second, third, and fourth quartiles was ≤ 15.56, 15.57 to 20.51, 20.51 to 27.78, and 27.82 to 67.82, respectively.

The general characteristics of study participants across the quartiles of DPI are demonstrated in Table 1. Individuals in the highest category of DPI were more likely to be female (*p*‐value < 0.001), younger (*p*‐value = 0.04), married (*p*‐value = 0.004), and highly educated (*p*‐value = 0.04). SBP (*p*‐value < 0.001), DBP (*p*‐value = 0.004), and TC level (*p*‐value = 0.03) had statistically significant differences among DPI categories, but they were not clinically significant. Dyslipidemia was also more frequent in subjects with the highest scores of DPI (P‐value = 0.01). Ethnicity varied significantly across DPI quartiles (*p*‐value < 0.001). There were no significant differences in BMI, waist circumference, physical activity, alcohol, TG, LDL, HDL, FBS levels, or the frequency of diabetes mellitus and hypertension across DPI quartiles.

**TABLE 1:** General characterizes of person with PCAD and healthy persons based on across quartiles of dietary phytochemical index.

Variables	Quartiles of DPI				
	Q1 (*N* = 790)	Q2 (*N* = 790)	Q3 (*N* = 791)	Q4 (*N* = 790)	*p* ^*a*^
Median (IQR)	12.52 (10.31–14.04)	17.94 (16.72–19.20)	23.78 (22.10–25.81)	32.82 (29.63–37.23)	
Age, years	54.35 ± 7.72	53.64 ± 7.62	53.68 ± 7.8	53.30 ± 7.49	0.04
Female *n* (%)	306 (38.7%)	311 (39.4%)	372 (47%)	438 (55.4%)	< 0.001
Ethnicity, *n* (%)					
Fars	344 (43.5%)	444 (56.2%)	459 (58%)	500 (63.3%)	< 0.001
Kurd	151 (19.1%)	70 (8.9%)	85 (10.7%)	46 (5.8%)	
Azari	69 (8.7%)	92 (11.6%)	47 (5.9%)	40 (5.1%)	
Lor	68 (8.6%)	40 (5.1%)	22 (2.8%)	26 (3.3%)	
Qashqaei	23 (2.9%)	34 (4.3%)	37 (4.7%)	34 (4.3%)	
Bakhtiari	38 (4.8%)	38 (4.8%)	61 (7.7%)	54 (6.8%)	
Arab	13 (1.6%)	12 (1.5%)	32 (4%)	32 (4.1%)	
Gilaki	83 (10.5%)	56 (7.1%)	45 (5.7%)	46 (5.8%)	
BMI, Kg/m^2^	27.97 ± 4.89	28.38 ± 4.94	28.36 ± 5.12	28.66 ± 5.64	0.06
Waist circumference, cm	98.4 ± 12.3	99.5 ± 12.7	99 ± 12.3	99.2 ± 13.0	0.95
Current smoking, *n* (%)	187 (23.7%)	181 (22.9%)	164 (20.7%)	116 (14.7%)	< 0.001
Physical activity, MET‐min/day	1951.54 ± 4078.45	1789.70 ± 3230.53	1923.30 ± 3487.22	1962.27 ± 4319.82	0.40
Alcohol	38 (4.8%)	35 (4.4%)	39 (4.9%)	21 (2.7%)	0.08
Married, *n* (%)	686 (86.8%)	719 (91%)	728 (92%)	710 (89.9%)	0.004
Education level, *n* (%)					
Undergraduate	719 (91%)	701 (88.7%)	703 (88.9%)	689 (87.2%)	0.04
Graduate	55 (7%)	74 (9.4%)	62 (7.8%)	81 (10.3%)	
Postgraduate	15 (1.9%)	15 (1.9%)	26 (3.3%)	20 (2.5%)	
SBP (mmHg)	120.6 ± 18.9	121.8 ± 18.4	122.9 ± 17.4	122.7 ± 17.8	< 0.001
DBP (mmHg)	76.7 ± 11.2	78.3 ± 11.2	78.8 ± 11.1	77.7 ± 11.4	0.004
Total cholesterol (mg/dL)	162.6 ± 46.7	165.3 ± 44.01	165.4 ± 44.2	164.1 ± 43.9	0.03
Triglyceride (mg/dL)	161.7 ± 90.6	156.2 ± 80.8	151.01 ± 79.6	148.7 ± 75.8	0.09
Low density lipoprotein (mg/dL)	89.1 ± 34.5	92.7 ± 35.7	91.5 ± 32.3	91.9 ± 32.8	0.15
High density lipoprotein (mg/dL)	42.8 ± 12.04	43.4 ± 11.08	43.4 ± 11.2	43.6 ± 11.1	0.49
Fasting blood sugar (mg/dL)	118.9 ± 56.4	114.9 ± 49.3	112.8 ± 49.6	115.07 ± 46.4	0.67
Dyslipidemia, *n* (%)	778 (98.5%)	776 (98.2%)	788 (99.6%)	785 (99.4%)	0.01
Hypertension, *n* (%)	350 (44.3%)	314 (39.7%)	353 (44.6%)	340 (43%)	0.08
Diabetes mellites, *n* (%)	239 (30.3%)	227 (28.7%)	217 (27.4%)	248 (31.4%)	0.06

*Note:* Data reported are mean ± SD.

Abbreviations: BMI, Body Mass Index; DBP, diastolic blood pressure; DPI, dietary phytochemical index; SBP, systolic blood pressure.

^a^ Chi‐square test was used for categorical variables and one‐way ANOVA for continuous variables.

The dietary intake of participants across quartiles of DPI is demonstrated in Table 2. Participants in the upper quartile of DPI had lower intake of energy, carbohydrate, protein, fat, monounsaturated fatty acid (MUFA), and calcium (*p*‐value < 0.001). In contrast, they had a higher intake of fiber, polyunsaturated fatty acid (PUFA), magnesium, potassium, vitamin E, and vitamin C (*p*‐value < 0.001). Intakes of fruits, vegetables, nuts, low‐fat dairy, legumes, and potato (*p*‐value < 0.001) were significantly higher in the upper quartile of DPI, while the intake of whole grains, refined grains, high‐fat dairy, and sugar‐sweetened beverages (SSB) (*p*‐value < 0.001) was significantly lower compared to the first quartile. Additionally, the intake of cholesterol, saturated fatty acid (SFA), and red and processed meat did not significantly differ across the DPI quartiles.

**TABLE 2:** Dietary intake of person with PCAD and healthy persons based on across quartiles of dietary phytochemical index.

Dietary intake	Quartiles of DPI				
	Q1 (*N* = 790)	Q2 (*N* = 790)	Q3 (*N* = 791)	Q4 (*N* = 790)	*p* ^a^
Energy, Kcal/d	2237.93 ± 25.5	2100.7 ± 23.9	1975.70 ± 23.4	1865.17 ± 23.6	< 0.001
**Nutrient intake**					
Carbohydrate (g/day)	302.7 ± 4.04	275.2 ± 3.6	245.4 ± 3.1	216.4 ± 3.1	< 0.001
Fat (g/day)	86.1 ± 1.3	83.5 ± 1.1	83.6 ± 1.1	85.9 ± 1.2	< 0.001
Protein (g/day)	87.4 ± 1.1	83.2 ± 1.03	81.4 ± 1.1	76.1 ± 1.1	< 0.001
Total fiber (g/day)	20.7 ± 0.3	21.1 ± 0.3	21.6 ± 0.3	22.0 ± 0.3	< 0.001
SFA (g/day)	34.1 ± 0.6	32.2 ± 0.5	30.7 ± 0.5	29.4 ± 0.5	0.29
PUFA (g/day)	20.8 ± 0.3	21.7 ± 0.3	23.0 ± 0.3	25.5 ± 0.4	< 0.001
MUFA (g/day)	26.4 ± 0.4	25.1 ± 0.3	24.9 ± 0.3	25.6 ± 0.4	< 0.001
**Food groups**					
Fruits (g/day)	161.3 ± 3.2	216.4 ± 3.3	242.3 ± 4.2	272.2 ± 4.5	< 0.001
Vegetables (g/day)	225.6 ± 3.6	272.8 ± 4.3	313.2 ± 5.7	367.4 ± 6.9	< 0.001
Whole grains (g/day)	165.1 ± 5.6	130.9 ± 4.8	111.0 ± 4.1	67.2 ± 2.8	< 0.001
Refined grains (g/day)	297.6 ± 6.4	253.2 ± 5.6	205.4 ± 4.4	177.5 ± 4.1	< 0.001
Red and process meat (g/day)	38.8 ± 1.6	35.2 ± 1.1	31.3 ± 1.2	26.0 ± 0.98	0.15
Nuts (g/day)	3.8 ± 0.2	5.5 ± 0.2	8.7 ± 0.3	14.6 ± 0.6	< 0.001
High‐fat dairy (g/day)	91.6 ± 3.9	91.4 ± 4.1	77.6 ± 3.7	56.2 ± 3.1	< 0.001
Low‐fat dairy (g/day)	248.2 ± 7.03	260.9 ± 6.9	272.4 ± 7.6	251.4 ± 7.3	< 0.001
Legumes (g/day)	37.3 ± 0.98	52.1 ± 1.30	65.2 ± 1.6	80.9 ± 2.5	< 0.001
SSB (g/day)	81.8 ± 5.4	55.2 ± 3.2	40.03 ± 2.6	28.6 ± 1.8	< 0.001
Potato (g/day)	18.7 ± 0.9	23.3 ± 1.1	22.5 ± 1.01	32.1 ± 1.5	< 0.001

*Note:* Data reported as mean ± SE.

Abbreviations: DPI, dietary phytochemical index; MUFA, monounsaturated fatty acid; PUFA, polyunsaturated fatty acid; SFA, saturated fatty acid; SSB, sugar sweetened beverage.

^a^ ANCOVA test was used for nutrients and food groups (all variables were adjusted for age, sex, and energy excepted for daily energy intake which was adjusted for age and sex).

**FIGURE 1:**
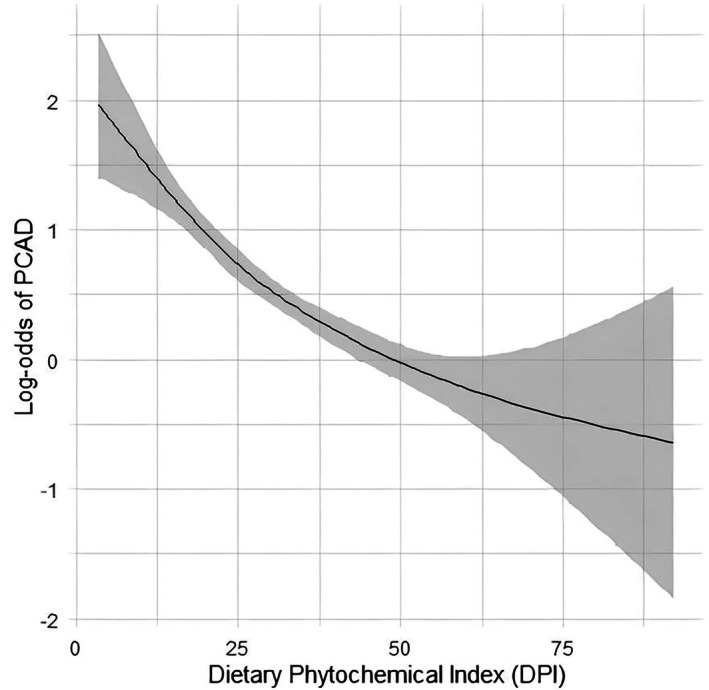
Restricted cubic spline regression model between the dietary phytochemical index and odds of PCAD. Cubic spline model: The four knots are the 0th, 25th, 50th, and 75th percentiles. The model adjusted for sex, age, daily energy intake, smoking, marital status, education, physical activity, alcohol, high‐fat dairy, low‐fat dairy, red and processed meat, potato, fiber, refined grain, sugar sweetened beverage, BMI, diabetes mellites, hypertension, dyslipidemia, and Aspirin.

Odds ratio and 95% confidence intervals (CI) of PCAD based on quartiles of DPI are demonstrated in Table 3 and Figure 1. A significant inverse link between DPI and PCAD was found in the crude model (Q4 vs. Q1: OR = 0.25, 95% CI = 0.20–0.31, *P* trend < 0.001). Adjustment for demographic and lifestyle variables in model 2 did not change the association (OR = 0.25, 95% CI = 0.20–0.33, *P* trend < 0.001). Furthermore, when accounting for the confounding effects of dietary intake, this association became stronger, and participants in the upper quartile of DPI had an 89% lower chance of PCAD compared to those in the lower quartile (OR = 0.11, 95% CI = 0.08–0.15, *P* trend < 0.001). This association was independent of mediators adjusted in the fully adjusted model (OR = 0.12, 95% CI = 0.08–0.17, *P* trend < 0.001). Restricted cubic spline regression between DPI and odds of PCAD is demonstrated in Figure 1. An inverse nonlinear dose–response association between DPI and risk of PCAD was found (*P* value < 0.001).

**TABLE 3:** Odds ratio and 95% confidence intervals of premature coronary artery disease across quartiles of dietary phytochemical index in the PCAD group.

Models	Quartiles of DPI				
	Q1 (*N* = 790)	Q2 (*N* = 790)	Q3 (*N* = 791)	Q4 (*N* = 790)	*P* trend^*a*^
Crude	1	0.55 (0.44–0.70)	0.39 (0.31–0.49)	0.25 (0.20–0.31)	< 0.001
Model 1	1	0.53 (0.41–0.68)	0.39 (0.30–0.50)	0.27 (0.21–0.34)	< 0.001
Model 2	1	0.52 (0.40–0.67)	0.37 (0.29–0.48)	0.25 (0.20–0.33)	< 0.001
Model 3	1	0.37 (0.28–0.49)	0.21 (0.16–0.29)	0.11 (0.08–0.15)	< 0.001
Model 4	1	0.52 (0.39–0.7)	0.37 (0.28–0.50)	0.25 (0.18–0.34)	< 0.001

*Note:* Data reported as OR (95% CI). Crude model: not adjusted. Model 1: adjusted for sex, age, and daily energy intake. Model 2: additionally, adjusted for smoking, marital status, education, physical activity, and alcohol. Model 3: additionally, adjusted for high‐fat dairy, low‐fat dairy, red and processed meat, potato, fiber, refined grain, and sugar sweetened beverage. Model 4: additionally, adjusted for BMI, diabetes mellites, hypertension, dyslipidemia, and Aspirin.

Abbreviations: DPI, dietary phytochemical index; PCAD, premature coronary artery disease.

^a^ *P* trend was obtained using logistic regression analysis.

## 4 Discussion

We observed a significant inverse association between DPI and PCAD in this case–control analysis. This relationship remained significant after adjustment for potential confounders. Participants with the highest score of DPI compared to those with the lowest score had 89% lower odds of PCAD.

Although previous research has demonstrated a negative relationship for the higher DPI and various cardiometabolic risk factors or metabolic syndrome (Iacoviello et al. 2018; Tong et al. 2020; Gamba, Pano, et al. 2023), our study is one of the first research studies which specifically explored the association between DPI and PCAD, as a cardiovascular event. CAD remains the leading cause of death globally (Wang et al. 2020; Okrainec et al. 2004), accounting for almost half of deaths among the Iranian population with a heavy burden on the health system and the families (Karimi‐Moonaghi et al. 2014; Mohammadifard et al. 2019). Therefore, preventing premature CVD would be of utmost importance. Besides metabolic risk factors, poor nutrition is a key lifestyle contributor to atherosclerosis progression (Poorzand et al. 2019; Osadnik et al. 2018; Sharma and Ganguly 2005). Improving dietary behaviors could potentially decrease both the onset and progression of PCAD (Le et al. 2024).

Our findings regarding phytochemicals are in agreement with prior research from the past several decades. The cardioprotective effect of various individual phytochemicals has been illustrated in several researches. In a cohort study involving 5209 adults, a negative relationship was reported between dietary lycopene intake and the incidence of CHD (Jacques et al. 2013). Moreover, higher serum lycopene levels were related with odds risk of CVD (Rissanen et al. 2001; Ito et al. 2006) and carotid artery intima‐media thickness in the eastern Finland population (Rissanen et al. 2000, 2003). Lycopene has been found to inhibit cardiac hypertrophy and protect against cardiovascular events by enhancing the activity of the antioxidant response element and promoting the expression of antioxidant‐related genes (D'Oria et al. 2020). Consistently, Street et al. demonstrated a negative relationship between serum β‐carotene levels and the risk of myocardial infarction (Street et al. 1991). Similar findings have been documented between flavonoid intake and incidence of CVD (Perez‐Vizcaino and Duarte 2010). Hertog et al. demonstrated that individuals in the highest tertial of dietary flavonoid intake had a 68% reduced risk of CVD compared to those in the lowest tertial (Hertog et al. 1993). In a prospective cohort research performed by Mukamal et al., it was found that the consumption of dietary flavonoids at moderate and heavy levels resulted in a 31% and 39% reduction in CVD risk, respectively (Mukamal et al. 2002). Increased consumption of lignans may also be associated with a reduced risk of coronary heart disease (CHD) development (Hu et al. 2021) and CVD (Xia et al. 2023).

The precise mechanism through which dietary phytochemicals exert protective effects against chronic disease remains incompletely understood (Shen et al. 2012). Previous research has demonstrated that an increased consumption of dietary phytochemicals can improve insulin resistance, modulate insulin production/secretion, safeguard pancreatic β cells, and promote the activity of insulin‐dependent glucose transporter 4 (GLUT‐4) (Gamba, Roa‐Diaz, et al. 2023; Mehranfar et al. 2022). Considering the influence of insulin resistance on the development of CAD (Bressler et al. 1996), it appears that DPI may have a protective effect on CAD by affecting serum insulin levels. Other potential mechanisms might be attributed to anti‐platelet (Demrow et al. 1995; Freedman et al. 2001), anti‐inflammatory (Carluccio et al. 2003) effects, and anti‐oxidant (Delshad Aghdam et al. 2021) properties of polyphenols besides their favorable impacts on endothelial function (Modena et al. 2002). Phytochemicals may have a positive impact by reducing inflammation through their effects on TNF‐alpha and hs‐CRP levels (Mazidi et al. 2020; Moradi et al. 2016). The presence of polyphenols in DPI has been demonstrated to reduce levels of TNF‐α (Moradi et al. 2016). Decreasing TNF‐α levels can lead to a decrease in leptin secretion and inflammatory markers (Liu et al. 2009). In addition, based on animal and human research, phytochemical supplementation reduced leptin secretion and serum levels (Murugan et al. 2021). Carotenoids, polyphenols, and flavonoids regulate hs‐CRP serum levels by diminishing and removing reactive oxygen species and free radicals production, inhibiting proinflammatory enzymes, protecting cells from inflammation and oxidative stress, and blocking neural signaling to lymphocytes B (Kim and Park 2022).

The study has notable strengths. Primarily, it is an inaugural study to examine the association between DPI and PCAD in a large sample size of a population consisting of different ethnicities. In this study, we utilized valid questionnaires to determine dietary intakes, and PCAD was identified using angiography results. Moreover, the roles of various confounders and mediators in the DPI–PCAD relationship were controlled. However, several limitations come with our study, which should be acknowledged during result interpretation. First, there may be recall bias due to the case–control study. Second, it is possible that some confounding variables were not measured. Third, given the retrospective case–control approach, it is not possible to confirm a causal association. Additionally, one important limitation of this study is that the control group consisted of hospital patients who, like the cases, had undergone coronary angiography. Consequently, the results cannot be generalized to the broader population and are applicable only to individuals undergoing angiographicprocedures.

## 5 Conclusion

The current analysis revealed a significant negative association between elevated DPI score and odds of PCAD, which remained robust after controlling for confounding variables. Therefore, advising to consume a diet with a higher amount of phytochemical rich foods may be a dietary approach to lower odds of PCAD. Nevertheless, further confirmation through longitudinal cohort studies and randomized clinical trials remains essential.

## Author Contributions

M.B.: writing – original draft, writing – review and editing, investigation, E.Z.: resources and executive manager, F.H.: conceptualization, methodology, supervision, writing – review and editing, investigation, N.M.: conceptualization, methodology, writing – review and editing, project administration, data curation, M.D.: analyze, A.K.F., N.A., N.S., M.L., S.G., A.S., M.C., A.A.: resources, N.S.: resources, project administration. All authors read the final draft and approved it.

## Ethics Statement

The protocol of the study was confirmed by the Ethics Committee of Isfahan University of Medical Sciences, Isfahan, Iran (IR.MUI.REC.1396.2.055).

## Consent

The consent to participate in the study was obtained at the beginning.

## Conflicts of Interest

The authors declare no conflicts of interest.

## Data Availability

The data that support the findings of this study are available from the corresponding author upon reasonable request.

## References

[fsn370943-bib-0001] Achari, V. , and A. K. Thakur . 2004. “Association of Major Modifiable Risk Factors Among Patients With Coronary Artery Disease—A Retrospective Analysis.” Journal of the Association of Physicians of India 52: 103–108. 15656042

[fsn370943-bib-0002] Acikgoz Pinar, A. , E. Yildiz , and K. Altundag . 2023. “Dietary Phytochemical Index and the Risk of Breast Cancer: A Case‐Control Study.” Nutrition and Cancer 75, no. 2: 482–487. 36104945 10.1080/01635581.2022.2122518

[fsn370943-bib-0003] Ahmadi, B. , A. Ramezani Ahmadi , M. Jafari , and N. Morshedzadeh . 2023. “The Association of Dietary Phytochemical Index and Nonalcoholic Fatty Liver Disease.” Food Science & Nutrition 11, no. 7: 4010–4019. 37457157 10.1002/fsn3.3389 PMC10345673

[fsn370943-bib-0004] Azadbakht, L. , N. Bellissimo , M. Darooghegi Mofrad , B. Guilani , and F. Siassi . 2019. “Association of Dietary Phytochemical Index and Mental Health in Women: A Cross‐Sectional Study.” British Journal of Nutrition 121, no. 9: 1049–1056. 30714542 10.1017/S0007114519000229

[fsn370943-bib-0005] Bahrampour, N. , A. Mirzababaei , D. Hosseininasab , F. Abaj , C. C. T. Clark , and K. Mirzaei . 2023. “High Intake of Dietary Phytochemical Index May Be Related to Reducing Risk of Diabetic Nephropathy: A Case–Control Study.” BMC Nutrition 9, no. 1: 14. 36647176 10.1186/s40795-023-00676-2 PMC9841724

[fsn370943-bib-0006] Bressler, P. , S. Bailey , M. Matsuda , and R. DeFronzo . 1996. “Insulin Resistance and Coronary Artery Disease.” Diabetologia 39: 1345–1350. 8933003 10.1007/s001250050581

[fsn370943-bib-0007] Carluccio, M. A. , L. Siculella , M. A. Ancora , et al. 2003. “Olive Oil and Red Wine Antioxidant Polyphenols Inhibit Endothelial Activation: Antiatherogenic Properties of Mediterranean Diet Phytochemicals.” Arteriosclerosis, Thrombosis, and Vascular Biology 23, no. 4: 622–629. 12615669 10.1161/01.ATV.0000062884.69432.A0

[fsn370943-bib-0008] Dehghani Firouzabadi, F. , A. Jayedi , E. Asgari , et al. 2021. “The Association of Dietary Phytochemical Index With Metabolic Syndrome in Adults.” Clinical Nutrition Research 10, no. 2: 161–171. 33987142 10.7762/cnr.2021.10.2.161 PMC8093085

[fsn370943-bib-0009] Delshad Aghdam, S. , F. Siassi , E. Nasli Esfahani , et al. 2021. “Dietary Phytochemical Index Associated With Cardiovascular Risk Factor in Patients With Type 1 Diabetes Mellitus.” BMC Cardiovascular Disorders 21, no. 1: 293. 34118879 10.1186/s12872-021-02106-2 PMC8199677

[fsn370943-bib-0010] Demrow, H. S. , P. R. Slane , and J. D. Folts . 1995. “Administration of Wine and Grape Juice Inhibits in Vivo Platelet Activity and Thrombosis in Stenosed Canine Coronary Arteries.” Circulation 91, no. 4: 1182–1188. 7850957 10.1161/01.cir.91.4.1182

[fsn370943-bib-0011] do Nascimento Nunes, M. C. 2008. “Impact of Environmental Conditions on Fruit and Vegetable Quality.” Stewart Postharvest Review 4: 1–14.

[fsn370943-bib-0012] D'Oria, R. , R. Schipani , A. Leonardini , et al. 2020. “The Role of Oxidative Stress in Cardiac Disease: From Physiological Response to Injury Factor.” Oxidative Medicine and Cellular Longevity 2020: 5732956. 32509147 10.1155/2020/5732956 PMC7244977

[fsn370943-bib-0013] Esposito, K. , R. Marfella , M. Ciotola , et al. 2004. “Effect of a Mediterranean‐Style Diet on Endothelial Dysfunction and Markers of Vascular Inflammation in the Metabolic Syndrome: A Randomized Trial.” Journal of the American Medical Association 292, no. 12: 1440–1446. 15383514 10.1001/jama.292.12.1440

[fsn370943-bib-0014] Expert Panel on Detection E , and Treatment of High Blood Cholesterol in A . 2001. “Executive Summary of the Third Report of the National Cholesterol Education Program (NCEP) Expert Panel on Detection, Evaluation, and Treatment of High Blood Cholesterol in Adults (Adult Treatment Panel III).” JAMA 285, no. 19: 2486–2497. 11368702 10.1001/jama.285.19.2486

[fsn370943-bib-0015] Freedman, J. E. , C. Parker Iii , L. Li , et al. 2001. “Select Flavonoids and Whole Juice From Purple Grapes Inhibit Platelet Function and Enhance Nitric Oxide Release.” Circulation 103, no. 23: 2792–2798. 11401934 10.1161/01.cir.103.23.2792

[fsn370943-bib-0016] Fung, T. T. , F. B. Hu , M. A. Pereira , et al. 2002. “Whole‐Grain Intake and the Risk of Type 2 Diabetes: A Prospective Study in Men.” American Journal of Clinical Nutrition 76, no. 3: 535–540. 12197996 10.1093/ajcn/76.3.535

[fsn370943-bib-0017] Gamba, M. , O. Pano , P. F. Raguindin , et al. 2023. “Association Between Total Dietary Phytochemical Intake and Cardiometabolic Health Outcomes—Results From a 10‐Year Follow‐Up on a Middle‐Aged Cohort Population.” Nutrients 15, no. 22: 4793. 38004187 10.3390/nu15224793 PMC10674839

[fsn370943-bib-0018] Gamba, M. , Z. M. Roa‐Diaz , P. F. Raguindin , et al. 2023. “Association Between Dietary Phytochemical Index, Cardiometabolic Risk Factors and Metabolic Syndrome in Switzerland. The CoLaus Study.” Nutrition, Metabolism, and Cardiovascular Diseases 33, no. 11: 2220–2232. 10.1016/j.numecd.2023.07.018 37598028

[fsn370943-bib-0019] Golzarand, M. , Z. Bahadoran , P. Mirmiran , S. Sadeghian‐Sharif , and F. Azizi . 2015. “Dietary Phytochemical Index Is Inversely Associated With the Occurrence of Hypertension in Adults: A 3‐Year Follow‐Up (The Tehran Lipid and Glucose Study).” European Journal of Clinical Nutrition 69, no. 3: 392–398. 25387902 10.1038/ejcn.2014.233

[fsn370943-bib-0020] Golzarand, M. , P. Mirmiran , Z. Bahadoran , S. Alamdari , and F. Azizi . 2014. “Dietary Phytochemical Index and Subsequent Changes of Lipid Profile: A 3‐Year Follow‐Up in Tehran Lipid and Glucose Study in Iran.” ARYA Atherosclerosis 10, no. 4: 203. 25258636 PMC4173317

[fsn370943-bib-0021] González, P. , T. Massardo , M. J. Jofré , et al. 2005. “201Tl Myocardial SPECT Detects Significant Coronary Artery Disease Between 50% and 75% Angiogram Stenosis.” Revista Española de Medicina Nuclear 24, no. 5: 305–311. 16194462 10.1157/13079281

[fsn370943-bib-0022] Hamedi‐Shahraki, S. , M.‐R. Jowshan , M.‐A. Zolghadrpour , F. Amirkhizi , and S. Asghari . 2023. “Dietary Phytochemical Index Is Favorably Associated With Oxidative Stress Status and Cardiovascular Risk Factors in Adults With Obesity.” Scientific Reports 13, no. 1: 7035. 37120685 10.1038/s41598-023-34064-4 PMC10148862

[fsn370943-bib-0023] Hemler, E. C. , and F. B. Hu . 2019. “Plant‐Based Diets for Cardiovascular Disease Prevention: All Plant Foods Are Not Created Equal.” Current Atherosclerosis Reports 21: 1–8. 30895476 10.1007/s11883-019-0779-5

[fsn370943-bib-0024] Hertog, M. G. , E. J. Feskens , D. Kromhout , P. Hollman , and M. Katan . 1993. “Dietary Antioxidant Flavonoids and Risk of Coronary Heart Disease: The Zutphen Elderly Study.” Lancet 342, no. 8878: 1007–1011. 8105262 10.1016/0140-6736(93)92876-u

[fsn370943-bib-0025] Hu, Y. , Y. Li , L. Sampson , et al. 2021. “Lignan Intake and Risk of Coronary Heart Disease.” Journal of the American College of Cardiology 78, no. 7: 666–678. 34384548 10.1016/j.jacc.2021.05.049 PMC8432598

[fsn370943-bib-0026] Iacoviello, L. , M. Bonaccio , G. Cairella , et al. 2018. “Diet and Primary Prevention of Stroke: Systematic Review and Dietary Recommendations by the Ad Hoc Working Group of the Italian Society of Human Nutrition.” Nutrition, Metabolism, and Cardiovascular Diseases 28, no. 4: 309–334. 10.1016/j.numecd.2017.12.010 29482962

[fsn370943-bib-0027] Ito, Y. , M. Kurata , K. Suzuki , N. Hamajima , H. Hishida , and K. Aoki . 2006. “Cardiovascular Disease Mortality and Serum Carotenoid Levels: A Japanese Population‐Based Follow‐Up Study.” Journal of Epidemiology 16, no. 4: 154–160. 16837766 10.2188/jea.16.154 PMC7603911

[fsn370943-bib-0028] Jacques, P. F. , A. Lyass , J. M. Massaro , R. S. Vasan , and R. B. D'Agostino Sr. 2013. “Relationship of Lycopene Intake and Consumption of Tomato Products to Incident CVD.” British Journal of Nutrition 110, no. 3: 545–551. 23317928 10.1017/S0007114512005417 PMC3710301

[fsn370943-bib-0029] Karimi‐Moonaghi, H. , M. Mojalli , and S. Khosravan . 2014. “Psychosocial Complications of Coronary Artery Disease.” Iranian Red Crescent Medical Journal 16, no. 6: e18162. 25068057 10.5812/ircmj.18162 PMC4102990

[fsn370943-bib-0030] Keaney, J. F., Jr. , M. G. Larson , R. S. Vasan , et al. 2003. “Obesity and Systemic Oxidative Stress: Clinical Correlates of Oxidative Stress in the Framingham Study.” Arteriosclerosis, Thrombosis, and Vascular Biology 23, no. 3: 434–439. 12615693 10.1161/01.ATV.0000058402.34138.11

[fsn370943-bib-0031] Khot, U. N. , M. B. Khot , C. T. Bajzer , et al. 2003. “Prevalence of Conventional Risk Factors in Patients With Coronary Heart Disease.” JAMA 290, no. 7: 898–904. 12928466 10.1001/jama.290.7.898

[fsn370943-bib-0032] Kim, C. , and K. Park . 2022. “Association Between Phytochemical Index and Inflammation in Korean Adults.” Antioxidants 11, no. 2: 348. 35204229 10.3390/antiox11020348 PMC8868203

[fsn370943-bib-0033] Le, A. , H. Peng , D. Golinsky , M. Di Scipio , R. Lali , and G. Paré . 2024. “What Causes Premature Coronary Artery Disease?” Current Atherosclerosis Reports 26: 189–203. 38573470 10.1007/s11883-024-01200-y

[fsn370943-bib-0034] Liu, J. , W. Shen , B. Zhao , et al. 2009. “Targeting Mitochondrial Biogenesis for Preventing and Treating Insulin Resistance in Diabetes and Obesity: Hope From Natural Mitochondrial Nutrients.” Advanced Drug Delivery Reviews 61, no. 14: 1343–1352. 19716392 10.1016/j.addr.2009.06.007

[fsn370943-bib-0035] Martínez‐González, M. A. , J. Salas‐Salvadó , R. Estruch , D. Corella , M. Fitó , and E. Ros . 2015. “Benefits of the Mediterranean Diet: Insights From the PREDIMED Study.” Progress in Cardiovascular Diseases 58, no. 1: 50–60. 25940230 10.1016/j.pcad.2015.04.003

[fsn370943-bib-0036] Mazidi, M. , N. Katsiki , E. S. George , and M. Banach . 2020. “Tomato and Lycopene Consumption Is Inversely Associated With Total and Cause‐Specific Mortality: A Population‐Based Cohort Study, on Behalf of the International Lipid Expert Panel (ILEP).” British Journal of Nutrition 124, no. 12: 1303–1310. 31434581 10.1017/S0007114519002150

[fsn370943-bib-0037] McCarty, M. F. 2004. “Proposal for a Dietary “Phytochemical Index”.” Medical Hypotheses 63, no. 5: 813–817. 15488652 10.1016/j.mehy.2002.11.004

[fsn370943-bib-0038] Mehranfar, S. , Y. Jalilpiran , H.‐S. Ejtahed , et al. 2022. “Association of Dietary Phytochemical Index With Cardiometabolic Risk Factors.” International Journal for Vitamin and Nutrition Research 93: 559–576. 35997240 10.1024/0300-9831/a000763

[fsn370943-bib-0039] Modena, M. G. , L. Bonetti , F. Coppi , F. Bursi , and R. Rossi . 2002. “Prognostic Role of Reversible Endothelial Dysfunction in Hypertensive Postmenopausal Women.” Journal of the American College of Cardiology 40, no. 3: 505–510. 12142118 10.1016/s0735-1097(02)01976-9

[fsn370943-bib-0040] Moghaddam, M. B. , F. B. Aghdam , M. A. Jafarabadi , H. Allahverdipour , S. D. Nikookheslat , and S. Safarpour . 2012. “The Iranian Version of International Physical Activity Questionnaire (IPAQ) in Iran: Content and Construct Validity, Factor Structure, Internal Consistency and Stability.” World Applied Sciences Journal 18, no. 8: 1073–1080.

[fsn370943-bib-0041] Mohammad, A. M. , H. I. Jehangeer , and S. K. Shaikhow . 2015. “Prevalence and Risk Factors of Premature Coronary Artery Disease in Patients Undergoing Coronary Angiography in Kurdistan, Iraq.” BMC Cardiovascular Disorders 15: 155. 26582255 10.1186/s12872-015-0145-7 PMC4650135

[fsn370943-bib-0042] Mohammadifard, N. , F. Haghighatdust , R. Kelishadi , et al. 2021. “Validity and Reproducibility of a Semi‐Quantitative Food Frequency Questionnaire for Iranian Adults.” Nutrition & Dietetics 78, no. 3: 305–314. 33786965 10.1111/1747-0080.12666

[fsn370943-bib-0043] Mohammadifard, N. , K. Mehrabani‐Zeinabad , F. Haghighatdoost , A. Mokdad , M. Naghavi , and N. Sarrafzadegan . 2019. “Ischemic Heart Disease Attributable to Dietary Risk Factors in the Middle East and North Africa (NAME) Region: An Analysis for the Global Burden of Disease Study 2019.” 10.48305/arya.2025.45265.3058 PMC12747298 41473580

[fsn370943-bib-0044] Moradi, S. , K. Mirzaei , A. A. Abdurahman , S. A. Keshavarz , and A. Hossein‐Nezhad . 2016. “Mediatory Effect of Circulating Vaspin on Resting Metabolic Rate in Obese Individuals.” European Journal of Nutrition 55: 1297–1305. 26058881 10.1007/s00394-015-0948-4

[fsn370943-bib-0045] Mukamal, K. J. , M. Maclure , J. E. Muller , J. B. Sherwood , and M. A. Mittleman . 2002. “Tea Consumption and Mortality After Acute Myocardial Infarction.” Circulation 105, no. 21: 2476–2481. 12034652 10.1161/01.cir.0000017201.88994.f7

[fsn370943-bib-0046] Murugan, D. D. , D. Balan , and P. F. Wong . 2021. “Adipogenesis and Therapeutic Potentials of Antiobesogenic Phytochemicals: Insights From Preclinical Studies.” Phytotherapy Research 35, no. 11: 5936–5960. 34219306 10.1002/ptr.7205

[fsn370943-bib-0047] Okrainec, K. , D. K. Banerjee , and M. J. Eisenberg . 2004. “Coronary Artery Disease in the Developing World.” American Heart Journal 148, no. 1: 7–15. 15215786 10.1016/j.ahj.2003.11.027

[fsn370943-bib-0048] Osadnik, T. , N. Pawlas , M. Lonnie , et al. 2018. “Family History of Premature Coronary Artery Disease (P‐CAD)‐A Non‐Modifiable Risk Factor? Dietary Patterns of Young Healthy Offspring of P‐CAD Patients: A Case‐Control Study (MAGNETIC Project).” Nutrients 10, no. 10: 1488. 30322041 10.3390/nu10101488 PMC6213507

[fsn370943-bib-0049] Panwar, R. B. , R. Gupta , B. K. Gupta , et al. 2011. “Atherothrombotic Risk Factors & Premature Coronary Heart Disease in India: A Case‐Control Study.” Indian Journal of Medical Research 134, no. 1: 26–32. 21808131 PMC3171913

[fsn370943-bib-0050] Patel, H. , S. Chandra , S. Alexander , J. Soble , and K. A. Williams . 2017. “Plant‐Based Nutrition: An Essential Component of Cardiovascular Disease Prevention and Management.” Current Cardiology Reports 19: 1–10. 28887684 10.1007/s11886-017-0909-z

[fsn370943-bib-0051] Perez‐Vizcaino, F. , and J. Duarte . 2010. “Flavonols and Cardiovascular Disease.” Molecular Aspects of Medicine 31, no. 6: 478–494. 20837053 10.1016/j.mam.2010.09.002

[fsn370943-bib-0052] Poorzand, H. , K. Tsarouhas , S. A. Hozhabrossadati , et al. 2019. “Risk Factors of Premature Coronary Artery Disease in Iran: A Systematic Review and Meta‐Analysis.” European Journal of Clinical Investigation 49, no. 7: e13124. 31038733 10.1111/eci.13124

[fsn370943-bib-0053] Rigi, S. , S. M. Mousavi , F. Shakeri , et al. 2022. “Dietary Phytochemical Index in Relation to Risk of Stroke: A Case‐Control Study.” Nutritional Neuroscience 25, no. 11: 2239–2246. 34311680 10.1080/1028415X.2021.1954291

[fsn370943-bib-0054] Rissanen, T. , S. Voutilainen , K. Nyyssönen , R. Salonen , and J. T. Salonen . 2000. “Low Plasma Lycopene Concentration Is Associated With Increased Intima‐Media Thickness of the Carotid Artery Wall.” Arteriosclerosis, Thrombosis, and Vascular Biology 20, no. 12: 2677–2681. 11116071 10.1161/01.atv.20.12.2677

[fsn370943-bib-0055] Rissanen, T. H. , S. Voutilainen , K. NyyssoÈnen , et al. 2001. “Low Serum Lycopene Concentration Is Associated With an Excess Incidence of Acute Coronary Events and Stroke: The Kuopio Ischaemic Heart Disease Risk Factor Study.” British Journal of Nutrition 85, no. 6: 749–754. 11430780 10.1079/bjn2001357

[fsn370943-bib-0056] Rissanen, T. H. , S. Voutilainen , K. Nyyssönen , R. Salonen , G. A. Kaplan , and J. T. Salonen . 2003. “Serum Lycopene Concentrations and Carotid Atherosclerosis: The Kuopio Ischaemic Heart Disease Risk Factor Study.” American Journal of Clinical Nutrition 77, no. 1: 133–138. 12499332 10.1093/ajcn/77.1.133

[fsn370943-bib-0057] Ross, R. 1999. “Atherosclerosis—An Inflammatory Disease.” New England Journal of Medicine 340, no. 2: 115–126. 9887164 10.1056/NEJM199901143400207

[fsn370943-bib-0058] Salas‐Salvadó, J. , N. Becerra‐Tomás , J. F. García‐Gavilán , M. Bulló , and L. Barrubés . 2018. “Mediterranean Diet and Cardiovascular Disease Prevention: What Do we Know?” Progress in Cardiovascular Diseases 61, no. 1: 62–67. 29678447 10.1016/j.pcad.2018.04.006

[fsn370943-bib-0059] Sarrafzadegann, N. , F. Ashrafi , M. Noorbakhsh , et al. 2009. “Association of Breast Artery Calcification With Coronary Artery Disease and Carotid Intima‐Media Thickness in Premenopausal Women.” Eastern Mediterranean Health Journal 15, no. 6: 1474–1482. 20218140

[fsn370943-bib-0060] Sharma, M. , and N. K. Ganguly . 2005. “Premature Coronary Artery Disease in Indians and Its Associated Risk Factors.” Vascular Health and Risk Management 1, no. 3: 217–225. 17319107 PMC1993956

[fsn370943-bib-0061] Shen, C.‐L. , B. J. Smith , D.‐F. Lo , et al. 2012. “Dietary Polyphenols and Mechanisms of Osteoarthritis.” Journal of Nutritional Biochemistry 23, no. 11: 1367–1377. 22832078 10.1016/j.jnutbio.2012.04.001

[fsn370943-bib-0062] Street, D. , G. Comstock , R. Salkeld , W. Schuep , and M. Klag . 1991. “A Population‐Based Case‐Control Study of the Association of Serum Antioxidants and Myocardial Infarction.” American Journal of Epidemiology 134: 719–720.

[fsn370943-bib-0063] Tong, T. Y. , P. N. Appleby , T. J. Key , et al. 2020. “The Associations of Major Foods and Fibre With Risks of Ischaemic and Haemorrhagic Stroke: A Prospective Study of 418 329 Participants in the EPIC Cohort Across Nine European Countries.” European Heart Journal 41, no. 28: 2632–2640. 32090257 10.1093/eurheartj/ehaa007 PMC7377582

[fsn370943-bib-0064] Vincent, H. K. , C. M. Bourguignon , and A. G. Taylor . 2010. “Relationship of the Dietary Phytochemical Index to Weight Gain, Oxidative Stress and Inflammation in Overweight Young Adults.” Journal of Human Nutrition and Dietetics 23, no. 1: 20–29. 19735350 10.1111/j.1365-277X.2009.00987.x PMC3641567

[fsn370943-bib-0065] Wang, L.‐J. , M.‐J. Liu , T.‐S. Zhai , et al. 2020. “Identification of U‐Shaped Curve Relation Between Proneurotensin and Risk of Coronary Artery Disease (CAD) in Patients With Premature CAD.” Nutrition, Metabolism, and Cardiovascular Diseases 30, no. 3: 483–491. 10.1016/j.numecd.2019.10.009 31926821

[fsn370943-bib-0066] World Health Organization . 1989. Measuring Obesity: Classification and Description of Anthropometric Data. Report on a WHO Consultation of the Epidemiology of Obesity, Warsaw, 21‐23 October 1987. World Health Organization.

[fsn370943-bib-0067] Xia, M. , Y. Chen , W. Li , and C. Qian . 2023. “Lignan Intake and Risk of Cardiovascular Disease and Type 2 Diabetes: A Meta‐Analysis of Prospective Cohort Studies.” International Journal of Food Sciences and Nutrition 74, no. 4: 501–509. 37282605 10.1080/09637486.2023.2220985

